# Crystal structure of dirubidium hydrogen citrate from laboratory X-ray powder diffraction data and DFT comparison

**DOI:** 10.1107/S2056989016020168

**Published:** 2017-01-01

**Authors:** Alagappa Rammohan, James A. Kaduk

**Affiliations:** a Atlantic International University, Honolulu, HI, USA; b Illinois Institute of Technology, Chicago, IL, USA

**Keywords:** crystal structure, powder diffraction, density functional theory, citrate, rubidium

## Abstract

The crystal structure of dirubidium hydrogen citrate, 2Rb^+^·HC_6_H_5_O_7_^2−^, has been solved and refined using laboratory X-ray powder diffraction data, and optimized using density functional techniques. The un-ionized carb­oxy­lic acid group forms helical chains of very strong hydrogen bonds (O⋯O ∼ 2.42 Å) along the *b* axis. The hy­droxy group participates in a chain of intra- and inter­molecular hydrogen bonds along the *c* axis. These hydrogen bonds result in corrugated hydrogen-bonded layers in the *bc* plane. The Rb^+^ cations are six-coordinate, and share edges and corners to form layers in the *ab* plane. The inter­layer contacts are composed of the hydro­phobic methyl­ene groups.

## Abstract 2

The crystal structure of dirubidium hydrogen citrate has been solved and refined using laboratory X-ray powder diffraction data, and optimized using density functional techniques.

## Chemical context   

In the course of a systematic study of the crystal structures of Group 1 (alkali metal) citrate salts to understand the conformational flexibility, ionization, coordination tendencies, and hydrogen bonding of the anion, we have determined several new crystal structures. Most of the new structures were solved using powder diffraction data (laboratory and/or synchrotron), but single crystals were used where available. The general trends and conclusions about the 16 new compounds and 12 previously characterized structures are being reported separately (Rammohan & Kaduk, 2017 ▸ ▸). Six of the new structures, *i.e.* NaKHC_6_H_5_O_7_, NaK_2_C_6_H_5_O_7_, Na_3_C_6_H_5_O_7_, NaH_2_C_6_H_5_O_7_, Na_2_HC_6_H_5_O_7_, and K_3_C_6_H_5_O_7_, have been published recently (Rammohan & Kaduk, 2016 ▸ *a* ▸,*b* ▸,*c* ▸,*d* ▸,*e* ▸; Rammohan *et al.*, 2016 ▸), and two additional structures, *i.e.* KH_2_C_6_H_5_O_7_ and KH_2_C_6_H_5_O_7_(H_2_O)_2_, have been communicated (Kaduk & Stern, 2016*a* ▸,*b* ▸) to the Cambridge Structural Database (Groom *et al.*, 2016 ▸).
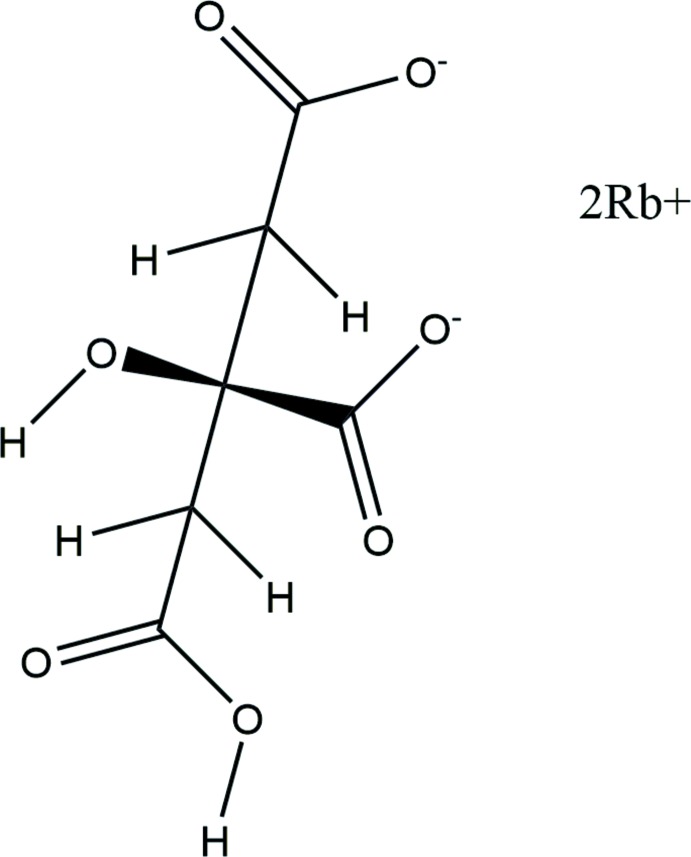


## Structural commentary   

**Figure 1:**
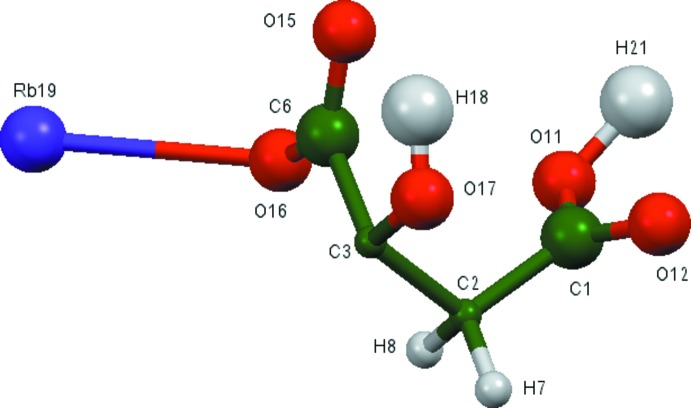
The asymmetric unit of the title compound, showing the atom numbering. The atoms are represented by 50% probability spheroids.

**Figure 2:**
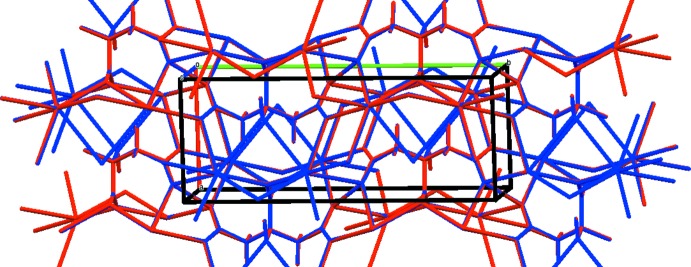
Comparison of the refined and optimized structures of dirubidium hydrogen citrate. The refined structure is in red and the DFT-optimized structure is in blue.

The asymmetric unit of the title compound is shown in Fig. 1 ▸. The r.m.s. deviation of the non-H atoms in the Rietveld refined and DFT-optimized structures is 0.052 Å (Fig. 2 ▸), and the maximum deviation is 0.083 Å, at atom C1. The good agreement between the two structures is strong evidence that the experimental structure is correct (van de Streek & Neumann, 2014 ▸). This discussion uses the DFT-optimized structure. Most of the bond lengths, bond angles, and torsion angles fall within the normal ranges indicated by a *Mercury Mogul Geometry Check* (Macrae *et al.*, 2008 ▸). The C1—C2—C3 angle of 111.1° is flagged as unusual [Z-score = 2.7; average = 114.3 (11)°]. The Z-score is the result of the low standard uncertainty on the average; the absolute difference of 3.2° is well within the expected range of such angles. The citrate anion occurs in the *trans*,*trans*-conformation, which is one of the two low-energy conformations of an isolated citrate. The central carboxyl­ate group and the hy­droxy group lie on the mirror plane. The central carboxyl­ate O15 atom and the terminal carb­oxy­lic acid O11 atom chelate to Rb19, and the central carboxyl­ate O16 atom and the terminal carb­oxy­lic acid O11 atom chelate to another Rb19. The Mulliken overlap populations and atomic charges indicate that the metal–oxygen bonding is ionic.

The Bravais–Friedel–Donnay–Harker (Bravais, 1866 ▸; Friedel, 1907 ▸; Donnay & Harker, 1937 ▸) morphology suggests that we might expect a platy morphology for dirubidium hydrogen citrate, with {020} as the principal faces. A 4th order spherical harmonic texture model was included in the refinement. The texture index was 1.078, indicating that preferred orientation was significant for this rotated flat plate specimen.

## Supra­molecular features   

**Figure 3:**
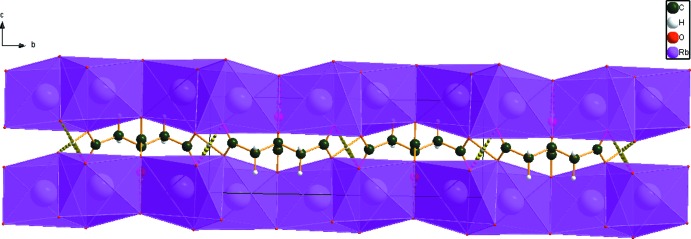
The crystal structure of dirubidium hydrogen citrate, viewed down the *a* axis.

The Rb cation is six-coordinate (bond-valence sum = 0.96). The coordination polyhedra share corners and edges to form layers in the *ab* plane (Fig. 3 ▸). The un-ionized terminal carb­oxy­lic acid forms a very strong symmetric hydrogen bond (Table 1 ▸). The Mulliken overlap population in the hydrogen-acceptor bond is 0.161 e. By the correlation in Rammohan & Kaduk (2017 ▸), this hydrogen bond accounts for 21.9 kcal mol^−1^ of crystal energy. The hy­droxy group participates in two hydrogen bonds to ionized central carboxyl­ate groups; one is intra­molecular with graph-set motif *S*(5), and the other is inter­molecular. These hydrogen bonds contribute 9.3 and 8.6 kcal mol^−1^ to the crystal energy.

## Database survey   

Details of the comprehensive literature search for citrate structures are presented in Rammohan & Kaduk (2017 ▸). A reduced cell search of the cell of dirubidium hydrogen citrate in the Cambridge Structural Database (Groom *et al.*, 2016 ▸) (increasing the default tolerance from 1.5 to 2.0%) yielded 12 hits, but limiting the chemistry to C, H, Rb, and O only resulted in no hits. The powder pattern is now contained in the the Powder Diffraction File (ICDD, 2016 ▸) as entry 00-063-1541.

## Synthesis and crystallization   

H_3_C_6_H_5_O_7_(H_2_O) (2.0768 g, 10.0 mmol) was dissolved in 10 ml deionized water. Rb_2_CO_3_ (10.0 mmol, 2.3170 g, Sigma–Aldrich) was added to the citric acid solution slowly with stirring. The resulting clear colorless solution was evaporated to dryness in an oven at 333 K.

## Refinement   

Crystal data, data collection and structure refinement details are summarized in Table 2 ▸. Entering 22 peaks (after manually applying a constant 2θ shift to approximate specimen displacement effects) into *ITO*/*CRYSFIRE* (Visser, 1969 ▸; Shirley, 2002 ▸) yielded a primitive monoclinic cell having *a* = 5.978, *b* = 15.096, *c* = 5.320 Å, β = 93.93°, *V* = 478.33 Å^3^, and *Z* = 2. Processing the pattern in *DASH3.2* (David *et al.*, 2006 ▸) suggested that the most probable space group was *P*2_1_, but no acceptable solution was found. A peak list was created from the results of a Le Bail fit using the REFLIST option in *GSAS*, and imported into *Endeavour1.7b* (Putz *et al.*, 1999 ▸). Using a citrate, two Rb atoms, and the O atom of a water mol­ecule as fragments yielded a successful structure solution. In the initial refinements, the water mol­ecule moved very close to one of the Rb atoms, and so was removed from the refinement.

**Figure 4:**
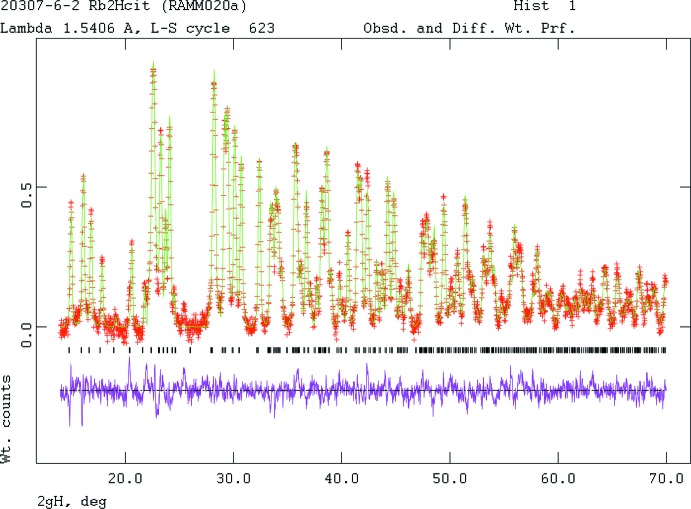
Rietveld plot for the refinement of dirubidium hydrogen citrate. The vertical scale is not the raw counts but the counts multiplied by the least-squares weights. This plot emphasizes the fit of the weaker peaks. The red crosses represent the observed data points and the green line is the calculated pattern. The magenta curve is the difference pattern, plotted at the same scale as the other patterns. The row of black tick marks indicates the reflection positions.

Pseudo-Voigt profile coefficients were as parameterized in Thompson *et al.* (1987 ▸) with profile coefficients for Simpson’s rule integration of the pseudo-Voigt function according to Howard (1982 ▸). The asymmetry correction of Finger *et al.* (1994 ▸) was applied, and microstrain broadening by Stephens (1999 ▸). The structure was refined by the Rietveld (Fig. 4 ▸) method using *GSAS*/*EXPGUI* (Larson & Von Dreele, 2004 ▸; Toby, 2001 ▸). All C—C and C—O bond lengths were restrained, as were all bond angles. The H atoms were included at fixed positions, which were recalculated during the course of the refinement using *Materials Studio* (Dassault Systemes, 2014 ▸). The *U*_iso_ of the atoms in the central and outer portions of the citrate were constrained to be equal, and the *U*_iso_ of the H atoms were constrained to be 1.3 times those of the atoms to which they are attached.

The structure was solved and initially refined in the space group *P*2_1_. The ADDSYM module of *PLATON* (Spek, 2009 ▸) suggested the presence of an additional centre of symmetry, and that the space group was *P*2_1_/*m*. Refinement in this space group yielded slightly better residuals (*R*_wp_ = 0.0277 and reduced χ^2^ = 3.3236, compared to *R*_wp_ = 0.0282 and χ^2^ = 3.454 for *P*2_1_), and we believe that *P*2_1_/*m* is the correct space group.

Stoichiometry requires one carb­oxy­lic acid proton per citrate. The space group *P*2_1_ is consistent with ordered asymmetric hydrogen bonds, while *P*2_1_/*m* is consistent with both disordered asymmetric hydrogen bonds or symmetric hydrogen bonds. Crystallographically, it would be difficult to distinguish these two possibilities, especially using X-ray powder diffraction data. DFT calculations on the asymmetric (*P*2_1_) and symmetric (*P*2_1_/*m*) hydrogen-bond models indicate that the symmetric model is 0.2 kcal mol^−1^ lower in energy. This difference is within the expected error range of such calculations. Since the crystallography strongly indicates the higher symmetry, we believe that the *P*2_1_/*m* model with symmetric hydrogen bonds is the best model for this structure.

## DFT calculations   

After the Rietveld refinement, a density functional geometry optimization (fixed experimental unit cell) was carried out using *CRYSTAL14* (Dovesi *et al.*, 2014 ▸). The basis sets for the C, H, and O atoms were those of Peintinger *et al.* (2012 ▸), and the basis set for Rb was that of Schoenes *et al.* (2008 ▸). The calculation was run on eight 2.1 GHz Xeon cores (each with 6 Gb RAM) of a 304-core Dell Linux cluster at IIT, used 8 *k*-points and the B3LYP functional, and took about 5 h. The *U*_iso_ from the Rietveld refinement were assigned to the optimized fractional coordinates.

## Supplementary Material

Crystal structure: contains datablock(s) RAMM020C_publ, ramm020c_DFT. DOI: 10.1107/S2056989016020168/vn2120sup1.cif

CCDC references: 1523504, 1523503

Additional supporting information: crystallographic information; 3D view; checkCIF report

## Figures and Tables

**Table 1:** Hydrogen-bond geometry (Å, °) for the DFT-optimized structure of dirubidium hydrogen citrate[Chem scheme1]

*D*—H⋯*A*	*D*—H	H⋯*A*	*D*⋯*A*	*D*—H⋯*A*
O11—H21⋯O11	1.209	1.209	2.418	180.0
O17—H18⋯O15	0.979	1.992	2.611	119.0
O17—H18⋯O16	0.979	1.992	3.216	148.6

**Table 2:** Experimental details

	Rietveld refinement	DFT optimization
Crystal data		
Chemical formula	2Rb^+^·HC_6_H_5_O_7_^2−^	2Rb^+^·HC_6_H_5_O_7_^2−^
*M*_r_	361.04	361.04
Crystal system, space group	Monoclinic, *P*2_1_/*m*	Monoclinic, *P*2_1_/*m*
Temperature (K)	300	300
*a*, *b*, *c* (Å)	5.97796 (17), 15.0960 (4), 5.32067 (19)	5.9780, 15.0961, 5.3207
β (°)	93.9341 (13)	93.9354
*V* (Å^3^)	479.02 (4)	478.99
*Z*	2	2
Radiation type	*K*α_1_, *K*α_2_, λ = 1.540629, 1.544451 Å	–
Specimen shape, size (mm)	Flat sheet, 24 × 24	–

Data collection		
Diffractometer	Bruker D2 Phaser	–
Specimen mounting	Normal sample holder	–
Data collection mode	Reflection	–
Data collection method	Step	–
θ values (°)	2θ_min_ = 5.042 2θ_max_ = 70.050 2θ_step_ = 0.020	–

Refinement		
*R* factors and goodness of fit	*R*_p_ = 0.021, *R*_wp_ = 0.028, *R*_exp_ = 0.015, *R*(*F* ^2^) = 0.0520, χ^2^ = 3.312	–
No. of parameters	49	–
No. of restraints	15	–
H-atom treatment	Only H-atom displacement parameters refined	–

Computer programs: *DIFFRAC.Measurement* (Bruker, 2009 ▸), *Endeavour* (Putz *et al.*, 1999 ▸), *GSAS* (Larson & Von Dreele, 2004 ▸), *DIAMOND* (Crystal Impact, 2015 ▸) and *publCIF* (Westrip, 2010 ▸).

## supplementary crystallographic information

### Crystal data

**Table:**

C_6_H_6_O_7_Rb_2_	*c* = 5.3207 Å
*M**_r_* = 361.04	β = 93.9354°
Monoclinic, *P*2_1_/*m*	*V* = 478.99 Å^3^
Hall symbol: -P 2yb	*Z* = 2
*a* = 5.9780 Å	*T* = 300 K
*b* = 15.0961 Å	

### Data collection

**Table:**

*h* = →	*l* = →
*k* = →	

### Fractional atomic coordinates and isotropic or equivalent isotropic displacement parameters (Å^2^)

**Table:**

	*x*	*y*	*z*	*U*_iso_*/*U*_eq_	
C1	0.28187	0.41704	0.52143	0.05270*	
C2	0.38508	0.33363	0.42243	0.01400*	
C3	0.27797	0.25000	0.53116	0.01400*	
C6	0.02276	0.25000	0.45797	0.05270*	
H7	0.56395	0.33136	0.47541	0.01820*	
H8	0.35795	0.33169	0.21841	0.01820*	
O11	0.10613	0.44671	0.38734	0.05270*	
O12	0.35379	0.45283	0.72015	0.05270*	
O15	−0.10430	0.25000	0.63801	0.05270*	
O16	−0.03680	0.25000	0.22772	0.05270*	
O17	0.31806	0.25000	0.79999	0.05270*	
H18	0.17127	0.25000	0.87091	0.06840*	
Rb19	−0.22414	0.10319	−0.03836	0.05980*	
H21	0.00000	0.00000	0.50000	0.07000*	

### Bond lengths (Å)

**Table:**

C1—C2	1.513	Rb19—O11^ii^	2.997
C1—O11	1.308	Rb19—O16	2.820
C1—O12	1.238	Rb19—O12^viii^	2.966
C2—C3	1.546	C3—C2^ii^	1.546
C2—H7	1.087	C3—C6	1.548
C2—H8	1.087	C3—O17	1.434
O11—Rb19^i^	3.115	C6—O15	1.263
O11—Rb19^ii^	2.997	C6—O16	1.252
O11—H21^iii^	1.209	O15—Rb19^ix^	2.926
O12—Rb19^iv^	2.879	O15—Rb19^x^	2.926
O12—Rb19^iii^	2.966	O16—Rb19^ii^	2.820
Rb19—O12^v^	2.879	O17—H18	0.979
Rb19—O15^vi^	2.926	H21—O11^ii^	1.209
Rb19—O11^vii^	3.115	H21—O11^viii^	1.209

Symmetry codes: (i) −*x*, *y*+1/2, −*z*; (ii) *x*, −*y*+1/2, *z*; (iii) −*x*, *y*+1/2, −*z*+1; (iv) *x*+1, −*y*+1/2, *z*+1; (v) *x*−1, −*y*+1/2, *z*−1; (vi) *x*, *y*, *z*−1; (vii) −*x*, *y*−1/2, −*z*; (viii) −*x*, *y*−1/2, −*z*+1; (ix) *x*, *y*, *z*+1; (x) *x*, −*y*+1/2, *z*+1.

### Hydrogen-bond geometry (Å, º)

**Table:**

*D*—H···*A*	*D*—H	H···*A*	*D*···*A*	*D*—H···*A*
O11—H21···O11	1.209	1.209	2.418	180.0
O17—H18···O15	0.979	1.992	2.611	119.0
O17—H18···O16	0.979	1.992	3.216	148.6
